# Reduced Ca^2+^ spark activity contributes to detrusor overactivity of rats with partial bladder outlet obstruction

**DOI:** 10.18632/aging.102855

**Published:** 2020-02-29

**Authors:** Ji Zheng, Hao Zhou, Mengjun Yang, Siji Song, Qiang Dai, Guangju Ji, Zhansong Zhou

**Affiliations:** 1 Department of Urology, Urological Surgery Research Institute, First Affiliated Hospital, Army Medical University (Third Military Medical University), Chongqing 400038, China; 2 Key Laboratory of Interdisciplinary Research, Institute of Biophysics, Chinese Academy of Sciences, Beijing 100049, China

**Keywords:** detrusor overactivity, Ca^2+^ spark, ryanodine receptor, FKBP12.6

## Abstract

We tested whether or not altered Ca^2+^ spark activity accounted for detrusor overactivity (DO) of Wistar rats after partial bladder outlet obstruction (PBOO). We constructed a DO model through PBOO and studied the Ca^2+^ spark activity of detrusor. By way of using confocal microscopy and the patch-clamp technique, Ca^2+^ sparks and spontaneous transient outward currents (STOCs) in detrusor myocytes were measured respectively. Our results indicated that Ca^2+^ spark activity and STOCs were significantly reduced in the DO detrusor myocytes compared to unafflicted control cells, and both of these had levels that were remarkably increased by applications of caffeine (10 μM), a RyR agonist, in DO myocytes. In addition, measures of detrusor contractions were also recorded by using freshly isolated detrusor strips. These results indicated that the spontaneous contraction of DO detrusor was significantly enhanced, and that the effect of caffeine (10 μM) upon detrusor contractions was reversed by applications of iberiotoxin (100 nM) which is a BK channel blocker. Western blotting (WB) analyses indicated that the levels of expression of ryanodine receptor type 2 (RyR2) and FK506 binding protein 12.6 (FKBP12.6) in bladder muscle were respectively decreased and increased in the samples from DO rats. Thus, we considered in the rat DO model wherein PBOO, the reduced Ca^2+^ spark activity in detrusor myocytes partly contributed to overactive detrusor contractions. The impaired Ca^2+^ spark activity may have resulted from decreased RyR2 expression and increased FKBP12.6 expression. Such novel findings in our research might help to provide means for better treatment outcomes for patients afflicted by bladder dysfunction.

## INTRODUCTION

Bladder dysfunction has a higher incidence in the elderly [1], and greatly affects the quality of life of afflicted [2]. This disease also results in costs that are significant in terms of medically oriented funding [3]. Natural aging processes can be commonly accompanied by the development and progression of dysfunctional conditions such as bladder hypertrophy [4] and bladder hypersensitivity [5]. Similar to these afflictions is partial bladder outlet obstruction (PBOO) which is also one of the most common causes of development and progression of detrusor overactivity (DO) [6, 7]. The condition of DO is associated with typically recognized symptoms such as increases in urinary frequency, urgency, and incontinence [8, 9]. DO induced by PBOO is thought to relate to changes in neurogenic and/or myogenic dynamics, detrusor innervation, cell-to-cell communication, and myocyte excitability [6–8]. The myogenic basis of DO is associated with an increased excitability and contractile activity of detrusor myocytes [8, 9].

Ryanodine receptors (RyRs) located within the sarcoplasmic reticulum (SR) are important modulators of excitation-contraction coupling in bladder myocytes [10, 11]. Spontaneous and localized increases in intracellular Ca^2+^ due to the opening of RyRs, which are visualized as Ca^2+^ sparks, activate large conductance of Ca^2+^ sensitive K+ channels (BK channels) that generate spontaneous transient outward currents (STOCs) [12, 13]. STOCs can shift the membrane potential towards less positive values, limit Ca^2+^ influx through L-type Ca^2+^ channels, and diminish global intracellular Ca^2+^ concentrations. It is through these mechanisms that RyRs/BK channels act as negative regulators of detrusor contraction [12, 14, 15].

In our previous study, we conducted detrusor strip experiments and found that measures for negative feedback regulation as related to RyRs/BK channels were weakened in DO muscle, which consequently resulted in spontaneous contractile overactivity [16]. Similarly oriented studies from within our own research lab [17] and other from others [18–20] have indicated that the levels of expression and functions of BK channels were found to have been decreased and diminished. These effects and findings provoked the idea that greater cell excitability could have been partly attributed to have accounted for the weakened measures of observed negative feedback regulation in DO muscle. Because the negative feedback regulation, presented as STOCs, is based on both RyRs and BK channels, and because RyR expression is significantly decreased in DO smooth muscle [16], possible consequential altered levels of Ca^2+^ spark activity caused by decrease of RyR expression may have also contributed to decreased STOCs and lead to DO.

Thus, in our study, we sought to establish rat PBOO models to imitate the changes of bladder dynamics after the onset of natural aging. We especially focused upon determinations of pathological changes of bladder dynamics after benign prostatic hyperplasia (BPH) in aging men. We expected that our data would demonstrate for the first time that decreased Ca^2+^ spark activity accounts for the previously recorded weakened measures of negative feedback regulation, thus contributing to overactive spontaneous contractions in DO muscle. We hypothesized that this reduction in Ca^2+^ spark activity may result from decreased expression of Ryanodine receptor 2 (RyR2) and increased expression of the RyR2 stabilizing protein, FK506 binding protein 12.6 (FKBP12.6), and sought to examine such factors. We hoped that our findings might provide a new attractive therapeutic target for clinically-based treatments of patients afflicted by DO.

## RESULTS

### Decreased STOCs activity in detrusor myocytes from DO rats

**Figure 1:**
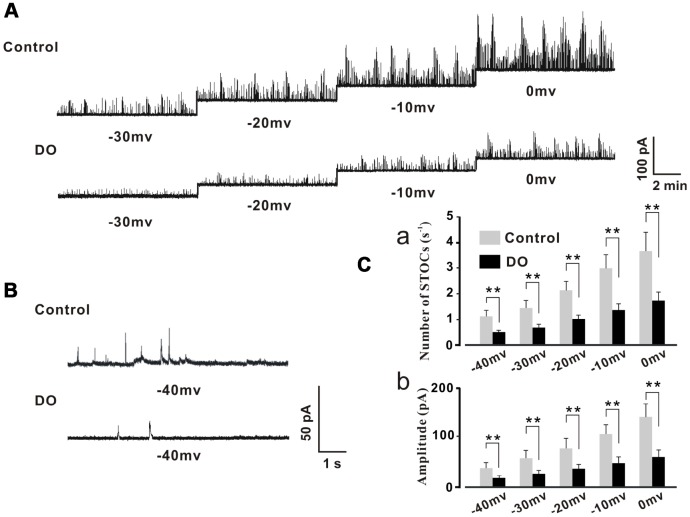
**Decreased STOC frequency and amplitude in detrusor myocytes of DO rats.** (**A**) Representative traces of STOCs in detrusor myocytes from control and DO rats. (**B**) Traces at a -40 mV holding potential from detrusor myocytes of control and DO rats. (**C**) Summarized data for measures of STOC frequency and amplitude. At all voltages, the frequencies (a) and amplitudes (b) of STOCs in DO rats were significantly lower than respective measures for rats in control groups. We used unpaired t tests for comparisons between groups. VS control *P<0.05, **P<0.01.

Our own previous study results indicated that RyRs/BK channel-related negative feedback regulation was weakened in DO muscle, resulting in the induction of spontaneous contractile overactivity [16]. Likewise, similarly oriented studies from our lab [9] and from other researchers [18, 19] have indicated and hypothesized that the decreased levels of expression and diminished functions of BK channels may partly account for the concomitant reduced measures of negative regulation. However, no attempts have thus far been made in order to test directly whether or not STOCs, which are a direct indicator of negative feedback regulation, are significantly decreased in detrusor myocytes of DO rats. Therefore, we first recorded measures for STOCs in freshly isolated detrusor myocytes by using the patch-clamp technique. Intact detrusor myocytes were patch-clamped at -40 mV or were progressively depolarized from -30 mV to 0 mV in 10-mV increments (Figure 1A).

The STOCs were subsequently successfully recorded in control and DO detrusor myocytes. We then were able to eliminate these STOCs by administration of iberiotoxin (100 nM) which was a BK channel blocker and this suggested that the detected STOCs were generated from BK channels (data not shown). The frequency and amplitude of STOCs were found to have been significantly decreased in DO detrusor myocytes (n/c=6/78) compared to control cells (n/c=6/95) spanning voltages from -40 mV to 0 mV (Figure 1A–1C). These data directly demonstrated the dramatic decrease in the frequencies and amplitudes of BK channel activities induced by Ca^2+^ sparks in the detrusor myocytes of DO rats [Number of STOCs (s^-1^), Ctrl vs DO, -40mv: 1.11 ± 0.24 vs 0.45 ± 0.09, -30mv: 1.42 ± 0.31 vs 0.66 ± 0.13, -20mv: 2.15 ± 0.34 vs 0.97 ± 0.15, -10mv: 2.99 ± 0.52 vs 1.36 ± 0.23, 0mv: 3.69 ± 0.73 vs 1.73 ± 0.34; Amplitude (pA), Ctrl vs DO, -40mv: 34.71 ± 10.96 vs 16.44 ± 4.57, -30mv: 55.71 ± 14.16 vs 25.12 ± 5.95, -20mv: 74.89 ± 18.73 vs 34.71 ± 8.68, -10mv: 103.21 ± 18.27 vs 45.21 ± 12.33, 0mv: 137.44± 25.11 vs 56.62 ± 14.62].

### Reduced Ca^2+^ spark properties and unaltered SR Ca^2+^ load in detrusor myocytes of DO rats

**Figure 2:**
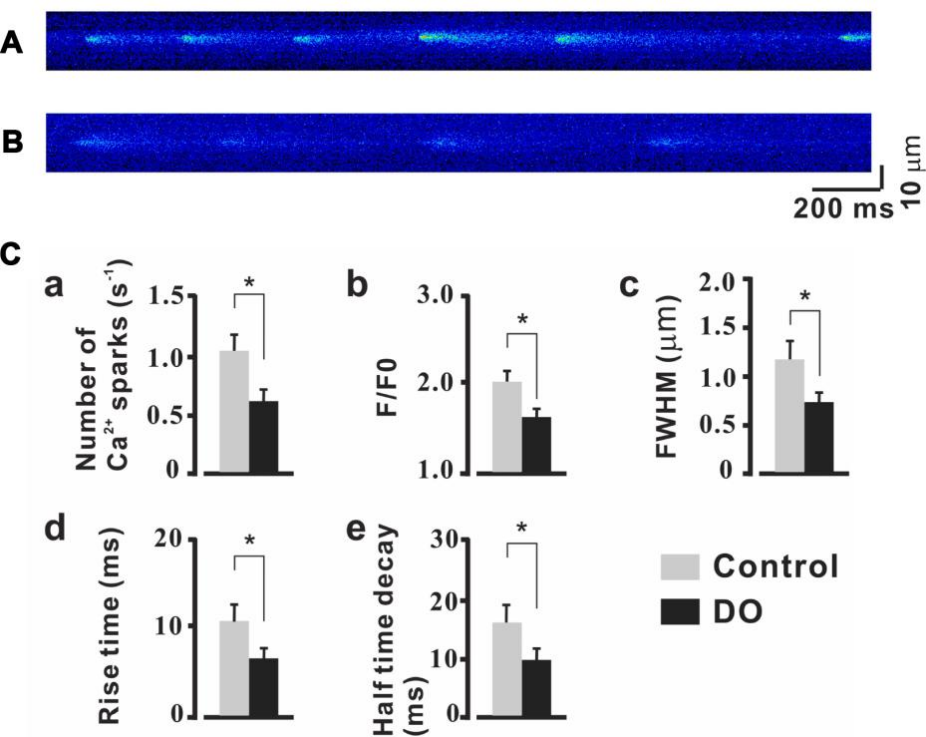
**Reduced properties of Ca2+ sparks in detrusor myocyte from DO model rats.** (**A**, **B**) Confocal linescans of representative Ca2+ sparks in detrusor myocyte from unafflicted control (**A**) and DO afflicted (**B**) rats. (**C**) Summary data for Ca2+ spark related properties. Ca2+ spark frequencies decreased in detrusor myocytes of DO afflicted samples (a). Similarly, F/F0 (b), FWHM (c), rise time (d), and half-time decay (e) all were found to have been significantly reduced in DO afflicted myocytes. We used unpaired t tests for comparisons between treatment groups. VS control *P<0.05, **P<0.01.

STOCs were fund to have been derived based upon both RyRs and BK channels and RyR expression was found to have been significantly decreased in DO smooth muscle [16], Thus, it is reasonable to hypothesize that alterations in the dynamics and levels of Ca^2+^ spark activity may also contribute to decreased STOCs. Already, such a finding has been noted in similar studies on cerebral artery smooth muscle cell [21, 22]. We thus tested measures of the properties of Ca2+ spark in detrusor myocytes. To record measures for Ca2+ sparks, we clamped the myocytes of control and DO detrusors at -40 mV. Examples of individual Ca2+ sparks were determined in high resolution and a summary of the properties of the resultant identified Ca2+ sparks are presented in Figure 2. As can be seen in Figure 2C, when compared to the measures for control cells (n/c=6/71), Ca2+ spark frequency was found to have decreased significantly in the detrusor myocytes of DO rats (n/c=6/85) (Figure 2Ca, Ctrl vs DO: 1.07 ± 0.15 vs 0.62 ± 0.11 s^-1^). Similarly, F/F0 (Figure 2Cb, Ctrl vs DO: 2.01 ± 0.12 vs 1.60 ± 0.10), FWHM (Figure 2Cc, Ctrl vs DO: 1.17 ± 0.22 vs 0.74 ± 0.11 μm), rise time (Figure 2Cd, Ctrl vs DO: 10.47 ± 1.79 vs 6.37 ± 1.16 ms), and half-time decay (Figure 2Ce, Ctrl vs DO: 16.61 ± 3.05 vs 10.03 ± 2.16 ms) were found to have had measures that were significantly reduced in DO cells. These data suggested that altered Ca2+ spark activity which existed may have accounted for the decreases in STOCs and increases in spontaneous contractions in DO muscle.

**Figure 3:**
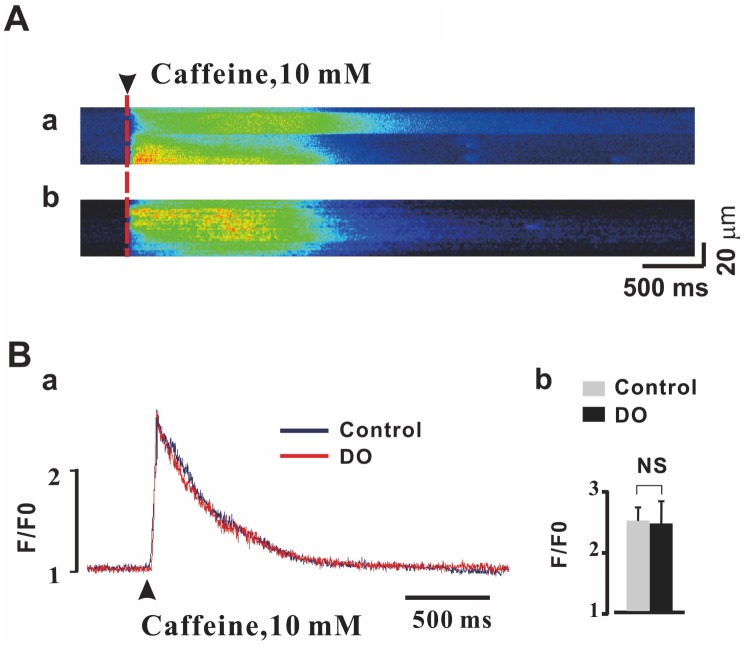
**Unaltered SR Ca2+ load in detrusor myocytes of DO model rats.** (**A**) Representative linescan images during 10 mM caffeine application in control (a) and DO (b) myocytes. (**B**) Peak amplitude of the caffeine-evoked fluorescence [Ca2+] i transient was not found to have been significantly different between DO afflicted and unafflicted control myocytes. We used unpaired t tests for comparisons between treatment groups. NS=Not significant.

Sarcoplasmic reticulum (SR) Ca2+ load is a major determinant of the activities and properties of Ca2+ spark. It is therefore plausible that a reduced SR Ca2+ load could contribute to concomitant reductions in Ca2+ spark properties described for the detrusor myocytes of DO rats. Thus, we measured whether or not global SR Ca2+ content was affected and affected these dynamics by rapid applications of caffeine (10 mM) [23]. As can be seen in Figure 3A and Figure 3B, no significant differences in values of the peak amplitude of the caffeine-evoked Ca2+ transients were observed between control (n/c=6/51) and DO (n/c=6/67) detrusor myocytes (Ctrl vs DO: 2.48 ± 0.24 vs 2.46 ± 0.38). Thus, we were able to rule out the possibility that the weaker measures of Ca2+ spark activity resulted from the reduced SR Ca2+ load in DO myocytes.

### Ca^2+^ sparks and STOCs were remarkably increased by caffeine and attenuated by ryanodine in both control and DO myocytes

**Figure 4:**
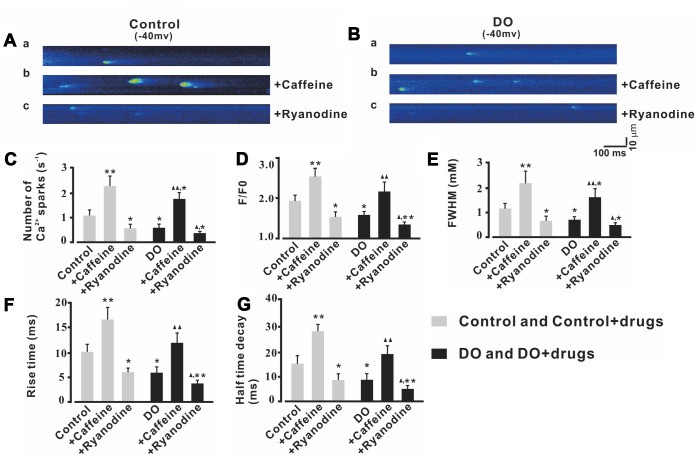
**Ca2+ sparks activity was increased by caffeine and was decreased by ryanodine in both unafflicted control and DO myocyte treatment groups.** (**A**, **B**) Representative Ca2+ sparks recorded in control (**A**) and DO (**B**) detrusor myocytes before and after exposure to10 μM caffeine (Ca2+ spark activator) (b) and before and after exposure to 10 μM ryanodine (Ca2+ spark inhibitor) (c). (**C**–**G**) provide summary data for Ca2+ spark frequency, F/F0, FWHM, rise time, and half-time decay of the control and DO destrusor unafflicted samples for treatments with caffeine (10 μM) and ryanodine (10 μM). To record the Ca2+ sparks, we clamped detrusor myocytes at -40 mV. We used one-way ANOVA for comparisons between groups. VS control, *P < 0.05, and **P < 0.01; VS DO, Δ P < 0.05, and ΔΔ P < 0.01.

Based on our findings and previous research, it was reasonable for us to test whether or not the effects of RyR regulators on Ca^2+^ sparks and STOCs of detrusor myocytes were significant before we explored whether or not reduced Ca^2+^ sparks contributed to the increased contractions in DO muscle. Caffeine is known to be able to lower the luminal Ca^2+^ threshold for RyR activation [24, 25] and even at as low as micromolar-level concentrations can cause an increase in both Ca^2+^ spark frequency and the number of active spark sites in the smooth muscle [26, 27]. Thus, we first examined Ca^2+^ sparks in the presence of a relatively low concentration (10 μM) of the RyR activator, caffeine. As can be seen in Figure 4, Ca^2+^ spark frequency (Figure 4C), F/F0 (Figure 4D), FWHM (Figure 4E), rise time (Figure 4F), and half-time decay (Figure 4G) were significantly increased by caffeine both in the control treatments and (n/c=6/57) (Figure 4Ab) and DO (n/c=6/71) (Figure 4Bb) detrusor myocytes. After application of ryanodine (10 μmol/L) which is an inhibitor of Ca^2+^ sparks, Ca^2+^ spark frequency (Figure 4C), F/F0 (Figure 4D), FWHM (Figure 4E), rise time (Figure 4F), and half-time decay (Figure 4G) were found to have been remarkably decreased in both control (n/c=6/51) (Figure 4Ac) and DO (n/c=6/63) (Figure 4Bc) detrusor myocytes [Number of Ca^2+^ sparks (s^-1^): Ctrl 1.06 ± 0.25, Ctrl + Caffeine 2.3 ± 0.42, Ctrl + Ryanodine 0.52 ± 0.17, DO 0.56 ± 0.15, DO + Caffeine 1.75 ± 0.28, DO + Ryanodine 0.33 ± 0.08; F/F0: Ctrl 1.91 ± 0.15, Ctrl + Caffeine 2.54 ± 0.2, Ctrl + Ryanodine 1.52 ± 0.12, DO 1.56 ± 0.09, DO + Caffeine 2.14 ± 0.25, DO + Ryanodine 1.31 ± 0.06; FWHM (mM): Ctrl 1.14 ± 0.20, Ctrl + Caffeine 2.15 ± 0.48, Ctrl + Ryanodine 0.61 ± 0.20, DO 0.66 ± 0.14, DO + Caffeine 1.56 ± 0.37, DO + Ryanodine 0.45 ± 0.09; Rise time (ms): Ctrl 10.18 ± 1.64, Ctrl + Caffeine 17.08 ± 2.46, Ctrl + Ryanodine 6.03 ± 0.89, DO 5.8 ± 1.27, DO + Caffeine 12.3 ± 1.94, DO + Ryanodine 3.65 ± 0.65; Half time decay (ms): Ctrl 15.2 ± 3.12, Ctrl + Caffeine 28.39 ± 2.70, Ctrl + Ryanodine 8.28 ± 2.51, DO 8.38 ± 2.41, DO + Caffeine 19.02 ± 3.15, DO + Ryanodine 4.55 ± 1.41].

**Figure 5:**
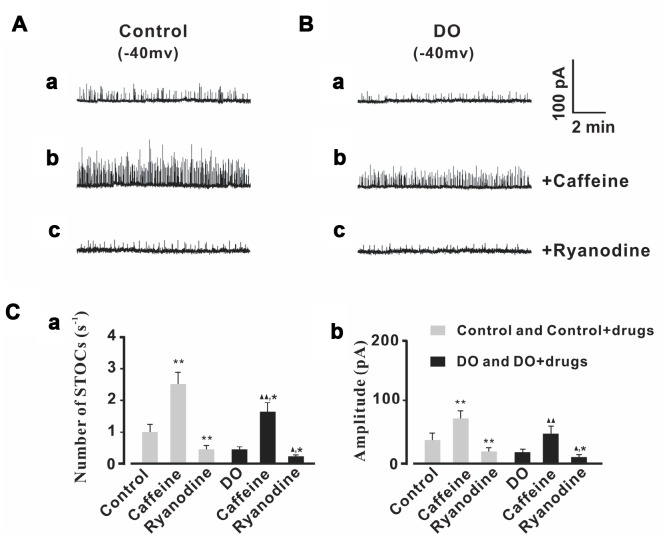
**STOCs were increased by caffeine and decreased by ryanodine in both control and DO myocytes.** (**A**, **B**) We recorded representative STOCs for control (**A**) and DO (**B**) detrusor myocytes before and after exposure to 10 μM caffeine (b) and before and after exposure to 10 μM ryanodine (c). Panels **C**a and **C**b provide summary data for frequencies and amplitudes of STOCs in unafflicted control and DO detrusor myocytes with regards to applications of caffeine (10 μM) and ryanodine (10 μM) (respectively). To record measures for STOCs, we clamped detrusor myocytes at -40 mV. We used one-way ANOVA for comparison between groups. VS control, *P < 0.05, and **P < 0.01; VS DO, Δ P < 0.05, and ΔΔ P < 0.01.

We then tested measures of the effects of Ca^2+^ spark regulators upon STOCs. As can be seen in Figure 5, the frequencies and amplitudes of STOCs were significantly decreased by application of ryanodine (10 μmol/L) in both the control treatments (n/c=6/41) and DO (n/c=6/63) detrusor myocytes. After application of caffeine (10 μmol/L), the frequencies and amplitudes of STOCs were found to have both significantly increased in both the control (n/c=6/47) and DO (n/c=6/56) detrusor myocytes. This suggested that Ca^2+^ spark activity was promoted and induced by caffeine and that such an application can restore the impaired negative feedback regulation (STOCs) in DO muscle [Number of STOCs (s^-1^): Ctrl 0.97 ± 0.22, Ctrl + Caffeine 2.51 ± 0.39, Ctrl + Ryanodine 0.43 ± 0.13, DO 0.42 ± 0.07, DO + Caffeine 1.64 ± 0.28, DO + Ryanodine 0.21 ± 0.05; Amplitude (pA): Ctrl 35.95 ± 10.54, Ctrl + Caffeine 70.67 ± 12.03, Ctrl + Ryanodine 16.82 ± 6.98, DO 17.01 ± 5.71, DO + Caffeine 45.4 ± 12.98, DO + Ryanodine 9.19 ± 3.79].

### Effects of Ca^2+^ spark regulators on detrusor contractions were reversed by a BK channel regulator in both control and DO detrusor strips

**Figure 6:**
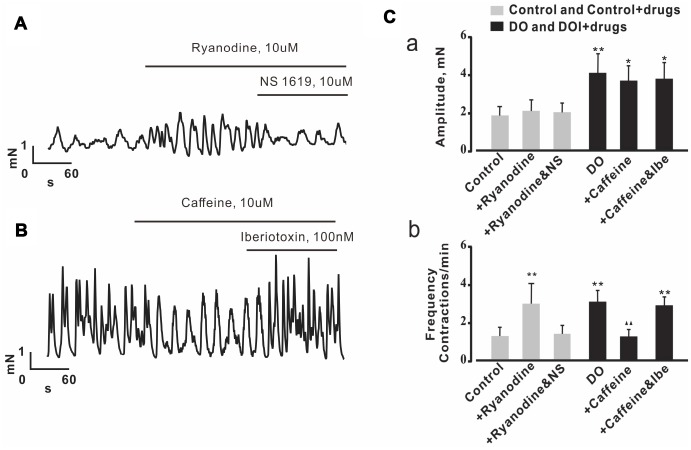
**Effects of Ca2+ spark regulators on detrusor contractions were reversed by the regulation of BK channels in both control and DO detrusor strips.** (**A**, **B**) Representative spontaneous contractions recorded in control (**A**) and DO (**B**) bladder strips after exposure to ryanodine (Ca2+ spark antagonist), caffeine (Ca2+ spark agonist), NS1619 (BK channel agonist) and iberiotoxin (BK channel antagonist). (**C**) Summary data for **A** and **B**, Frequency of spontaneous contractions was significantly increased by application of ryanodine in examinations of control detrusor strips and was decreased by caffeine in examinations of DO afflicted detrusor strips. The effects of ryanodine and caffeine on detrusor contractions were found to have been reversible by way of application of NS1619 or of iberiotoxin, respectively based upon examinations of the data from control and DO detrusor strips. We used one-way ANOVA for comparisons between treatment groups. VS control, *P < 0.05, and **P < 0.01; VS DO, Δ P < 0.05, and ΔΔ P < 0.01. NS=Not significant.

In the detrusor strip experiments, we used Ca^2+^ spark regulators to investigate whether or not decreased measures of Ca^2+^ spark activity contributed to increased spontaneous contractions in DO muscle. Consistent with the results from our own similarly oriented previous research [16], ryanodine was found to have significantly increased measures of the frequency of spontaneous contractions derived from control detrusor strips (Figure 6A, Figure 6Cb). Interestingly, the effect of ryanodine on detrusor contractions was abolished by way of application of the BK channel activator, NS1619 (10 μM) (Figure 6A, Figure 6Cb) (n/c=6/21). We then next explored measures of the effects of caffeine on data for spontaneous contractions derived from DO detrusor strips. In our results for this assessment using DO bladder strips, we found that the frequency of spontaneous contractions was significantly inhibited by 10 μM caffeine (Figure 6B, Figure 6Cb). This result suggested that there was the promotion of Ca^2+^ spark activity that was induced by caffeine, and that such an application could restore the impairment of negative feedback regulation in DO muscle. Similarly, based upon our results from the use of DO detrusor strips, we found that the effect of caffeine on spontaneous contractions was reversed by application of iberiotoxin (100 nM) which is a BK channel blocker (n/c=6/25) [Amplitude (mN): Ctrl 1.83 ± 0.47, Ctrl + Caffeine 2.04 ± 0.58, Ctrl + Ryanodine 1.97 ± 0.48, DO 4.07 ± 0.98, DO + Caffeine 3.66 ± 0.75, DO + Ryanodine 3.79 ± 0.85; Frequency (Contractions/min): Ctrl 1.22 ± 0.45, Ctrl + Caffeine 2.86 ± 1.07, Ctrl + Ryanodine 1.31 ± 0.44, DO 2.98 ± 0.59, DO + Caffeine 1.18 ± 0.36, DO + Ryanodine 2.8 ± 0.42].

### RyR2 expression was decreased and FKBP12.6 expression was increased in detrusors of DO rats

**Figure 7:**
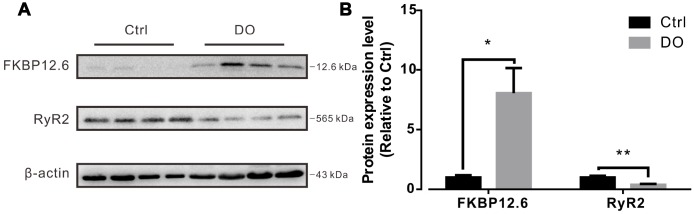
**Decreased expression of ryanodine receptor-2 (RyR2) and increased expression of FKBP12.6 in detrusors of rats with DO.** (**A**) Representative results from Western blotting analyses of RyR2, FKBP12.6, and GADPH (used as an internal control) from detrusors of unafflicted control rats and from DO afflicted rats. (**B**) Summarizations of levels of expression of RyR2 and FKBP12.6 proteins, given as ratios to beta actin. We used one-way ANOVA for comparisons between treatment groups. VS control, *P < 0.05, and **P < 0.01.

The RyR2 subtype is known to be the most dominantly expressed RyR isoform in the detrusors of smooth muscle [21, 23, 28]. FKBP12.6 specifically binds with RyR2 and stabilizes the closed state of RyR2 in cardiomyocytes and plays the same role in smooth muscle [23, 28, 29]. Previous research has indicated that animals that are both deficient in FKBP12.6 [22, 30] compared to the same species which contrastingly overexpress FKBP12.6 [24, 31] exhibited a more significant level of alterations in Ca^2+^ spark related properties. Because overexpression of FKBP12.6 has been reported to decrease Ca^2+^ spark activity in cardiac myocytes [24, 31], it is reasonable to hypothesize that increased levels of expression of FKBP12.6 or decreased levels of expression of RyR2 may both contribute to a reduction in Ca^2+^ spark activity in the myocytes of DO muscle. By way of using Western blotting, and when we normalized our findings to beta actin, we found that levels of expression of proteins of FKBP12.6 protein were significantly increased and that levels of expression of RyR2 were remarkably decreased in DO afflicted animals (n=4) compared with control animals (n=4) (Figure 7, Ctrl vs DO, FKBP12.6: 1.00 ± 0.19 vs 8.05 ± 2.10, RyR: 1.00 ± 0.13 vs 0.38 ± 0.08).

## DISCUSSION

Relevant epidemiological studies have confirmed that the prevalence of overactive bladder increases in populations with concomitant advancing age [3]. Especially for elderly males, obstructions of the lower urinary tract caused by BPH will ultimately lead to symptoms of resultant from DO including such as urinary urgency and urinary incontinence [32]. In recent experiments which examined measures of bladder function of aging mice, our own results also indicated that with the increase of age, the measures of urinary frequency and urinary incontinence in mice increased and these were the main symptom of DO [Supplementary Figure 1, void spots (3 hours), 7 months: 13.00 ± 2.210 N=16, 10 months: 41.50 ± 8.687 N=14, 13 months: 127.5 ± 27.31 N=10, 16 months: 242.0 ± 32.31 N=7, 20 months: 249.2 ± 22.29 N=5]. Accordingly, in this study, we also used PBOO to establish the DO model after we induced lower urinary tract obstruction in the rat. Our associated findings presented novel evidence that suggested that decreased Ca^2+^ spark activity contributed to weakened resultant measures for STOCs, which partly lead to resultant overactive contractions in DO muscle.

As STOCs are a known reliable direct measures and indicator of negative feedback regulation related to RyRs/BK channels, we first sought to investigate whether or not STOCs were significantly decreased in the detrusor myocytes of DO rats. Our novel results directly showed that there was a dramatic decrease in the frequencies and amplitudes of STOCs in the detrusor myocytes of DO rats (Figure 1). These results are similar to descriptions of measures of decreased STOC activity found in cerebral artery smooth cells sampled from a subarachnoid hemorrhage (SAH) [21] as well as are similar to findings which examined diabetic cell-based modeling dynamics [22].

Because the generation of STOCs is reliant upon both RyRs and BK channels [12], it is plausible that STOCs can result from the functional alteration of BK channels, RyRs, or from alterations of both these factors. In examinations of vascular SMCs of rats with diabetes [33] or with genetic hypertension [34], impairment of BK channel activity has been reported to have contributed to decreased measures related to STOCs. Similarly oriented studies from our own lab [17] and other researchers [18, 19] have likewise indicated that the decreased levels of expression and reduced activity of BK channels could have been attributed to have partly accounted for the weakened negative feedback regulation in the DO detrusor. According to findings from both Koide et al. [21] and Rueda et al. [22], in examinations of cerebral artery smooth cells there were reduced measures for STOCs and this many have also similarly resulted from decreased Ca2+ spark activity. Our own previous similar research results indicated that levels of expression of RyR were significantly decreased in DO smooth muscle [16], which in sum all strongly suggested that decreased Ca2+ spark activity potentially contributed to the reduced STOCs in detrusor myocytes of DO rats. Likewise, in the present study, we found that Ca2+ spark activity was significantly decreased in the detrusor myocytes of DO rats (Figure 2), which suggested that functional Ca2+ spark sites were remarkably attenuated in detrusor myocytes of DO rats. This also suggested that decreased STOCs may have been partly resultant as a consequence of reduced Ca2+ spark activity in the detrusor myocytes of DO rats.

It has previously been reported that inducing a decrease in SR Ca2+ load reduces Ca2+ spark properties in detrusor myocytes [23] and in vascular myocytes [35]. It is reasonable therefore to postulate that a reduced SR Ca2+ load could have contributed to the reduction in Ca2+ spark properties in detrusor myocytes of DO rats. Consistent with results from a previous study, we did not find a significant decrease in the SR Ca2+ load in DO detrusor myocytes (Figure 3), which suggested that the decreased activity in Ca^2+^ sparks did not result from inducing alterations in the loads of SR Ca^2+^.

To investigate whether or not decreased Ca2+ spark activity contributed to increased spontaneous contractions in DO, we used both an agonist and antagonist of RyR in Ca2+ spark, STOC, and further detrusor strip-based experiments. We used applications of caffeine, and used an RyR activator. When these are used at very low concentrations (μM - 1 mM) they cause an increase in Ca2+ spark activity but also induce a robust Ca2+ release that depletes Ca2+ stores when used in high concentrations (>5 mM) [36]. Similar to findings from previous studies [21], we found that application of treatments with caffeine increased the Ca2+ spark activity in both control and DO groups, which suggested that potential functional Ca2+ spark sites can be activated when needed in detrusor myocytes (for both groups). After application of ryanodine (10 μM) which is an inhibitor of Ca2+ sparks, we found that Ca2+ spark activity was significantly decreased in both control and DO detrusor myocytes (Figure 4). In our STOC-based experiments, we found that the frequencies and amplitudes of STOCs were significantly increased by application of caffeine for both the control and DO detrusor myocytes treatment groups. This result suggested that the increased measures of Ca2+ spark activity were likely to have been induced by applications of caffeine, and that such applications may be used to help to restore impairments in negative feedback regulation (STOCs) in DO muscle (Figure 5).

In the detrusor strip-based experiments, our findings appeared to be consistent with results from our own similarly oriented previous research [16]. For example, ryanodine was found to significantly induce an increase in the frequencies of spontaneous contractions in assessments wherein we used control detrusor strips, and we were able to abolish the effect of ryanodine on detrusor contractions by application of a BK channel activator, NS1619 (Figure 6). These data suggested that RyRs appear to play significant roles in the dynamics of negative feedback regulation of detrusor contraction frequency. Further, our results suggested these roles are influenced by the activity of BK channels, and these findings are likewise consistent with previous studies [12, 15, 16, 37]. In this study we found that an application of 10 μM of caffeine induced decreases in the frequencies of spontaneous contractions in DO detrusor strips (Figure 6). These results suggested that the promotion of Ca2+ spark activity induced by caffeine can be also be used to help to restore the impaired negative feedback regulation of detrusor contractions in DO muscle. Interestingly, in our methodology in which we used DO detrusor strips, we found that the effect of caffeine upon the frequency of spontaneous contractions was eliminated by counter application of iberiotoxin (Figure 6). These data further support the idea that decreased levels of activity of Ca2+ spark accounts for subsequently increased spontaneous contractions in DO muscle. In whole, our study results indicated that decreased Ca2+ spark activity was attributed to have partially accounted for weakened measures of data for STOCs by which there was also increased measures for contractions in DO muscle.

Spark sites comprise a variable number of RyRs, and functional spark sites require associated activities of a critical number of RyRs for Ca2+ sparks to complete all associated functions [22, 36]. Previous studies have indicated that decreased measures of Ca2+ activity can result from reduced levels of expression of RyR2 in examinations of cardiac myocytes [38] as well as for examinations of vascular [39] and artery smooth cells [21, 22]. Consistent with our findings of reduced levels of expression of RyR in our own previous research [16], in the present study we found that the levels of expression of RyR2 in detrusors were significantly decreased after DO. In addition to reduced levels of expression of RyR2, our findings indicated that there was increased levels of expression of FKBP12.6. This molecule is a pivotal regulator of RyR2, as well as can also contribute to Ca^2+^ spark reduction in both cardiac myocytes [24, 31] as well as artery smooth cell [23]. Similar to findings from a similarly oriented previous study [21] on artery smooth cells, our results indicated that levels of expression of FKBP12.6 proteins were significantly increased in DO bladders (Figure 7). Only 10% to 20% of endogenous myocyte RyR2s were found to have had concomitantly associated FKBP12.6, however, virtually all myocyte FKBP12.6 is RyR2-bound (because of its very high affinity) [40].

Therefore, decreased levels of expression of RyR2 and increased measures of FKBP12.6 may have both contributed to the observed DO-induced reduction in Ca^2+^ spark activity. However, similar to findings from a study by Koide et al. [21], the relative contributions of measures of decreased levels of expression of RyR2 versus for measures of increased levels of expression of FKBP12.6 to the induction of reduced Ca^2+^ spark activity in DO detrusor myocytes still require further exploration. Another important issue that may be worthy of further investigation is whether or not defective crosstalk between RyRs and BK channels is an important contributor to the major dynamics and mechanistics underlying DO.

## CONCLUSIONS

We conclude that the decreased Ca^2+^ spark activity in detrusor myocytes partly contributed to the observations of overactive spontaneous contractions in DO muscle. The decreased measures of Ca^2+^ spark activity may have resulted from a reduction in the levels of expression of RyR2 and an increase in the levels of expression of FKBP12.6. Thus, our novel approach and findings in this study can help to provide new ideas, applications, and better treatment outcomes for patients afflicted by DO caused by aging.

## MATERIALS AND METHODS

Female Wistar rats aged 3-5 months obtained from the Laboratory Animal Center of the Third Military Medical University were used in our studies. This study, all aspects of design and implementation, and experiments were approved by and were performed according to the guidelines set by the Laboratory Animal Welfare and Ethics Committee of the Third Military Medical University, Chongqing, China.

### PBOO and filling cystometry

We used PBOO and cystometry for group classification as has been reported upon previously [16, 17]. Measures of data for our study rats were assessed by using cystometry and after 6 weeks of treatments with PBOO. The urinary bladder was exposed, gently freed from adhering tissues, emptied and then cannulated with a plastic cannula. The free tip of the bladder cannula was connected to a pressure transducer and was connected to a peristaltic pump to allow for continuous infusion of a warm furacillin solution (37 °C) into the urinary bladder at a flow-rate of 10 mL/h. During infusion, intravesical pressure and voided urine volume were recorded continuously using a computer interface whereby: DO was confirmed when spontaneous contractions appeared in the recordings of intravesical pressure and when the amplitude of at least one contraction was >15 cmH2O.

### Isometric tension recording of strips

Strips from DO afflicted bladders and from normal unafflicted bladders were prepared as has been reported upon previously [16]. One end of the strip was attached to a stationary metal hook, and the other was connected with a silk suture to a force-displacement transducer such as in order to measure values for isometric contractility. We equilibrated urinary bladder strips at a resting load of 2 mN in physiological saline with periodic changes of the bathing fluid. Only strips which demonstrated reproducible contractile responses to 2-3 applications of 60 mM KCl were included for further study and analyses. Iberiotoxin (IBTX) and NS-1619 were purchased from Sigma-Aldrich (USA).

### Cell isolation

Single detrusor myocytes were prepared as previously described [23]. Briefly, we removed the urinary bladder and dissected this organ when placed in an ice-cold oxygenated Ca^2+^-free solution. The detrusor muscle was minced and incubated for 20 min at 37 °C in dissociation solution containing 1 mg/mL dithioerythreitol, 1 mg/mL papain, and 1 mg/mL bovine serum albumin (BSA). Then, the partially digested tissue was transferred to a solution containing 1 mg/mL collagenase type II (Worthington Biochemical), 1 mg/mL BSA, and 100 μM Ca^2+^. Cells were concentrated by use of low-speed centrifugation, were washed with fresh medium, resuspended, and finally were stored at 4 °C**.**

### Patch-Clamp recording

We recorded values for membrane currents at room temperature using whole-cell voltage clamp methods as previously described [23]. To examine STOCs, we clamped cells at -40 mV, or used steps up in voltage from -30 to 0 mV in 10-mV increments, such as in order to examine voltage dependence. We used an intracellular solution which was composed of (in mM) 130 KCl, 1.8 MgCl_2_, 1.0 Na_2_ATP, 0.05 CaCl_2_, and 0.1 EGTA (pH 7.3). The extracellular solution was composed of (mM) 137 NaCl, 5.4 KCl, 1.8 CaCl_2_, 1.0 MgCl_2_, 10 glucose, and 10 HEPES (pH 7.4). The currents were filtered at 500 Hz and we digitized results at 2 kHz.

### Measurement of Ca2+ fluorescence

We incubated myocytes with 10 μM Fluo-4 AM (Molecular Probes) for 10 min at room temperature and transferred samples into recording chambers mounted on an inverted microscope (IX81; Olympus) as previously described [28]. We allowed cells to adhere to the bottom of the recording chamber for 15 min and we then perfused the samples with extracellular solution for 40 min. Cells were then excited with application of a 488 nm light wavelength spectrum from a krypton/argon laser, and linescan images were collected using a laser scanning confocal head attached to an inverted microscope (IX81; Olympus). We captured and obtained linescans at intervals of 1.33 or 0.833 ms per line. Images were processed and analyzed using MATLAB 7.1 software (MathWorks). In order to accurately record measures related to Ca^2+^ sparks, we clamped detrusor myocytes at -40 mV. In SR Ca^2+^ load experiments, we used short applications of 10 mM of caffeine (Sigma) by way of pressure ejections from a glass pipette in order to estimate measures of SR Ca^2+^ content as has also been similarly previously described. All experiments were conducted at room temperature.

### Western blotting

Proteins were prepared as has been previously reported [16], and were then separated by using 4% to 20% acrylamide gradient gels. Proteins were then electrophoretically transferred onto a nitrocellulose-based membrane. The membranes were blocked for 2 h with the use and application of Tris-buffered saline-Tween 20 (TBST) containing 5% BSA at room temperature. The membrane was then incubated overnight with primary antibodies at 4 °C. After incubation with the appropriate secondary antibody for 1 hour at RT, we made measures of signal detection by way of using enhanced chemiluminescence detection solution 1 and 2 (1:1) (ECL; Millipore) and used this to measure levels of expression of RYR2, FKBP12.6, and GAPDH. The following antibodies were used: anti-RyR2 mouse monoclonal antibody (clone C3-33, 1:200; ABR, Golden, CO, USA), anti-FKBP12.6 rabbit polyclonal antibody (1:1000, Santa Cruz, CA, USA), anti-GAPDH goat polyclonal antibody (1:200,000, Santa Cruz, CA, USA), peroxidase-conjugated sheep antimouse IgG (1:2500, Santa Cruz, CA, USA), IRdye700-conjugated donkey anti-rabbit IgG and IRdye800-conjugated goat anti-mouse IgG (1:2500, Santa Cruz, CA, USA).

### Data analysis

We completed image processing and data analyses with customized software written in MATLAB and following methods that have been previously described [see: 23, 28]. Ca^2+^ sparks were counted manually and were also counted via determinations from a spark-counting software algorithm in order to help us to objectively verify the results. We made measurements for variables associated with STOC rise time and peak current by personal visual observations and from the raw values of current recordings. The results were expressed and characterized as the mean ± SEM where applicable. We assessed measures of statistical significance of differences in treatment groups by using unpaired t-tests or one-way ANOVA when appropriate.

## Supplementary Material

Supplementary Figure 1
