# Supplementary material for: Reduced Ca2+ spark activity contributes to detrusor overactivity of rats with partial bladder outlet obstruction
Source: Aging (Albany NY). 2020 Feb 29;12(5):4163–77. doi: 10.18632/aging.102855 (PMC7093189; doi:10.18632/aging.102855)
Supplement: Supplementary Figure 1 [file aging-12-102855-s001..pdf]

SUPPLEMENTARY FIGURE

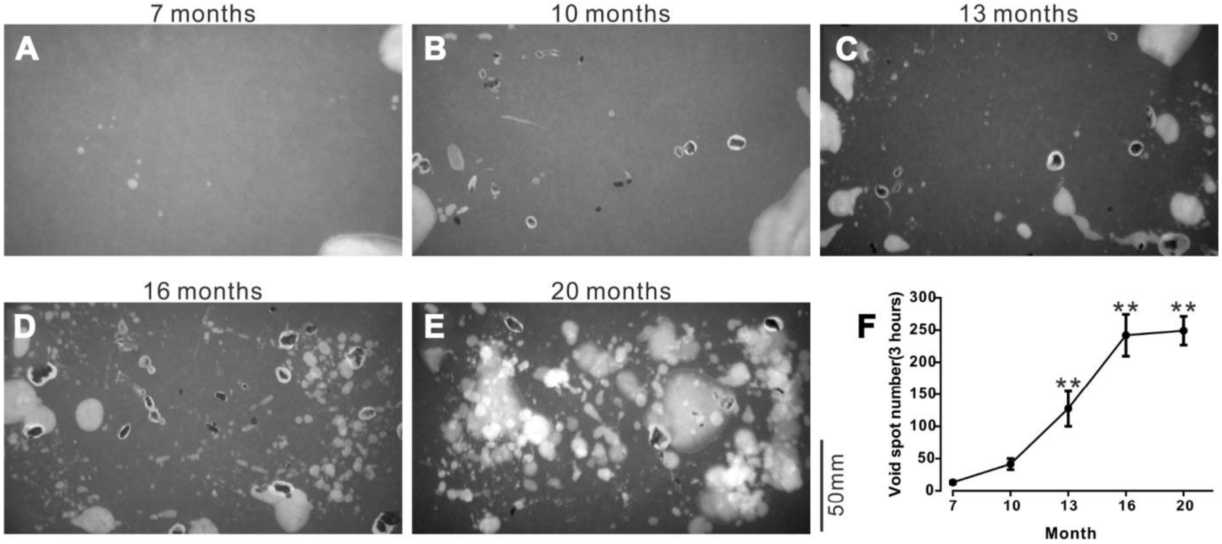

Supplementary Figure 1. The void spots of mice were found to have increased concomitantly with an increase in age. (**A**–**E**) are void spots of 7 month, 10 month, 13 month, 16 month, and 20 month aged mice respectively. (**F**) Summary data of (**A**–**E**). We used one-way ANOVA for comparisons between treatment groups. VS 7 months, **P < 0.01.
